# Regulation of miRNAs by Snail during epithelial-to-mesenchymal transition in HT29 colon cancer cells

**DOI:** 10.1038/s41598-019-39200-7

**Published:** 2019-02-15

**Authors:** Patrycja Przygodzka, Izabela Papiewska-Pająk, Helena Bogusz-Koziarska, Ewelina Sochacka, Joanna Boncela, M. Anna Kowalska

**Affiliations:** Institute of Medical Biology, PAS, 106 Lodowa Street, 93232 Lodz, Poland

## Abstract

Epithelial-to-mesenchymal transition (EMT) in cancer cells, represents early stages of metastasis and is a promising target in colorectal cancer (CRC) therapy. There have been many attempts to identify markers and key pathways induced throughout EMT but the process is complex and depends on the cancer type and tumour microenvironment. Here we used the colon cancer cell line HT29, which stably overexpressed Snail, the key transcription factor in early EMT, as a model for colorectal adenocarcinoma cells with a pro-metastatic phenotype. We investigated miRNA expression regulation during that phenotypic switching. We found that overexpression of Snail in HT29 cells triggered significant changes in individual miRNA levels but did not change the global efficiency of miRNA processing. Snail abundance repressed the expression of miR-192 and miR-194 and increased miR-205, let-7i and SNORD13 levels. These identified changes correlated with the reported transcriptomic alterations in Snail-overexpressing HT29 cells. We also investigated how Snail affected the miRNA content of extracellular vesicles (EVs) released from HT29 cells. Our data suggest that the presence of Snail significantly alters the complex mRNA/miRNA interactions in the early steps of metastasis and also has an impact on the content of EVs released from HT29 cells.

## Introduction

According to the newest projections published by the American Cancer Society, despite significant overall reductions in colorectal cancer (CRC) incidence and mortality, there is a need for further efforts to advance therapies on the early stage of cancer and metastasis development^1^. To escape from the primary tumour site and to form metastatic lesions, epithelial cancer cells must acquire a more migratory phenotype to overcome several anatomical barriers. The process of conversion of the epithelial cell phenotype towards a more mesenchymal phenotype (EMT process) is considered to be an initial and critical for metastasis. Although there are many concepts of how EMT is modified in cancer, (reviewed by Gurzu *et al*.^2^), the mechanism of EMT is still incompletely elucidated and there are many conflicting results published. During cancer EMT, there is a dynamic modulation of the interplay between transcription factors, gene expression and miRNAs (microRNAs /small non-coding RNAs)^3^. Thus, the understanding of the molecular interactions during the phenotype switch towards more mesenchymal and invasive cells has become important for future therapeutic strategies.

We previously performed a global transcriptomic and phenotypic characteristic of the HT29 colorectal adenocarcinoma cell line affected by the transcription factor Snail (*Snai1*), a core regulator of the early stages of the epithelial phenotype conversion that initiates metastasis. We have shown that upregulation of Snail in HT29 cells results in an incomplete phenotype conversion, up to the intermediate epithelial state^4^. We and others have found that enhanced Snail expression is associated with a more aggressive phenotype, poorer clinical outcomes and more frequent distant metastases^5,6^, and we proposed neuromedin U as a potential new biomarker of EMT^4^. Thus, we posit that Snail-overexpressing HT29 cells represent the phenotype of cancer cells at the leading edge of the primary tumour, with a more invasive phenotype, that are prepared for escape from the primary niche.

Since their discovery, vast amounts of miRNAs have been described and a growing number of correlations between miRNA expression and the development of cancer and cancer metastasis has been reported^7,8^. Because of their high tissue specificity and stability, specific miRNAs detected in colorectal cancer tissue, stool, serum or plasma are suggested as good candidates for diagnostic, prognostic, and predictive biomarkers of CRC and its progression^9,10^. However there are limitations to this approach. Conflicting results are reported for the functions of specific miRNAs in CRC^11^ that can be tumour site or cell phenotype dependent. Although the presence of a defined group of miRNAs has been shown to provide high diagnostic accuracy, for instance in breast cancers^12^, knowledge of the molecular mechanisms and regulatory networks leading to miRNA regulation over the course of EMT induction in CRC is still limited^13^.

Here, we focused on changes in miRNA expression at the very beginning of EMT that is mediated by Snail in HT29 cells. We demonstrated that downregulation of metastasis suppressors, miR-192 and miR-194, can be associated with Snail binding to the pri-miR-192/194 precursor promoter. We also showed that Snail induces the upregulation of miR-205 and let-7i, which is reflected in the content of extracellular vesicles released from Snail-overexpressing HT29 cells.

## Results

### Snail has no effect on the global efficiency of miRNA processing

To identify miRNAs engaged in Snail-activated regulatory pathways at the early stages of EMT in CRC, we used a previously generated and characterized model of colon cancer HT29 cells stably overexpressing Snail. We used clones of HT29 cells with various Snail overexpression levels: control clone HT29-pcDNA, HT29-Snail clone 3 (HT29-Snail-3) with moderate Snail overexpression and clones HT29-Snail-8 and -17 with high levels of Snail protein^4^.

miRNA expression profiling was performed on HT29-pcDNA, and HT29-Snail-3 and -8 clones. Clones 3 and 8 differ significantly in Snail expression level, and miRNA analysis was performed to expand our previously published transcriptomic studies performed on the same two clones^4^.

**Figure 1:**
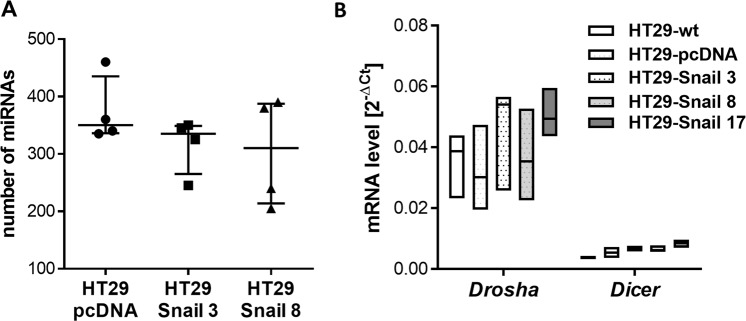
Snail upregulation in HT29 cells has no effect on the global efficiency of miRNA processing. (**A**) Plot showing the number of miRNAs detectable above the background threshold for each sample (out of a total of 2080 possible microRNAs and SNORDs). (**B**) The relative expression of *Drosha* and *Dicer* genes in HT29 clones; n = 4.

Figure 1A shows a summary of the present calls for each sample, i.e., the number of miRNAs detected above the background threshold. The obtained number of present calls is within the expected range. It is known^14,15^ that under certain conditions, the global efficiency of miRNA processing can be regulated, but our experiment showed that there were no significant differences in the number of detectable miRNAs between HT29-Snail and control clones. Moreover, Snail overexpression did not alter the relative expression levels of the key miRNA processing enzymes *Drosha* and *Dicer* (Fig. 1B). Thus we excluded modifications of the miRNA processing efficiency by Snail in our model.

### Changes in the expression of specific miRNAs induced by Snail in colon cancer cells

**Figure 2:**
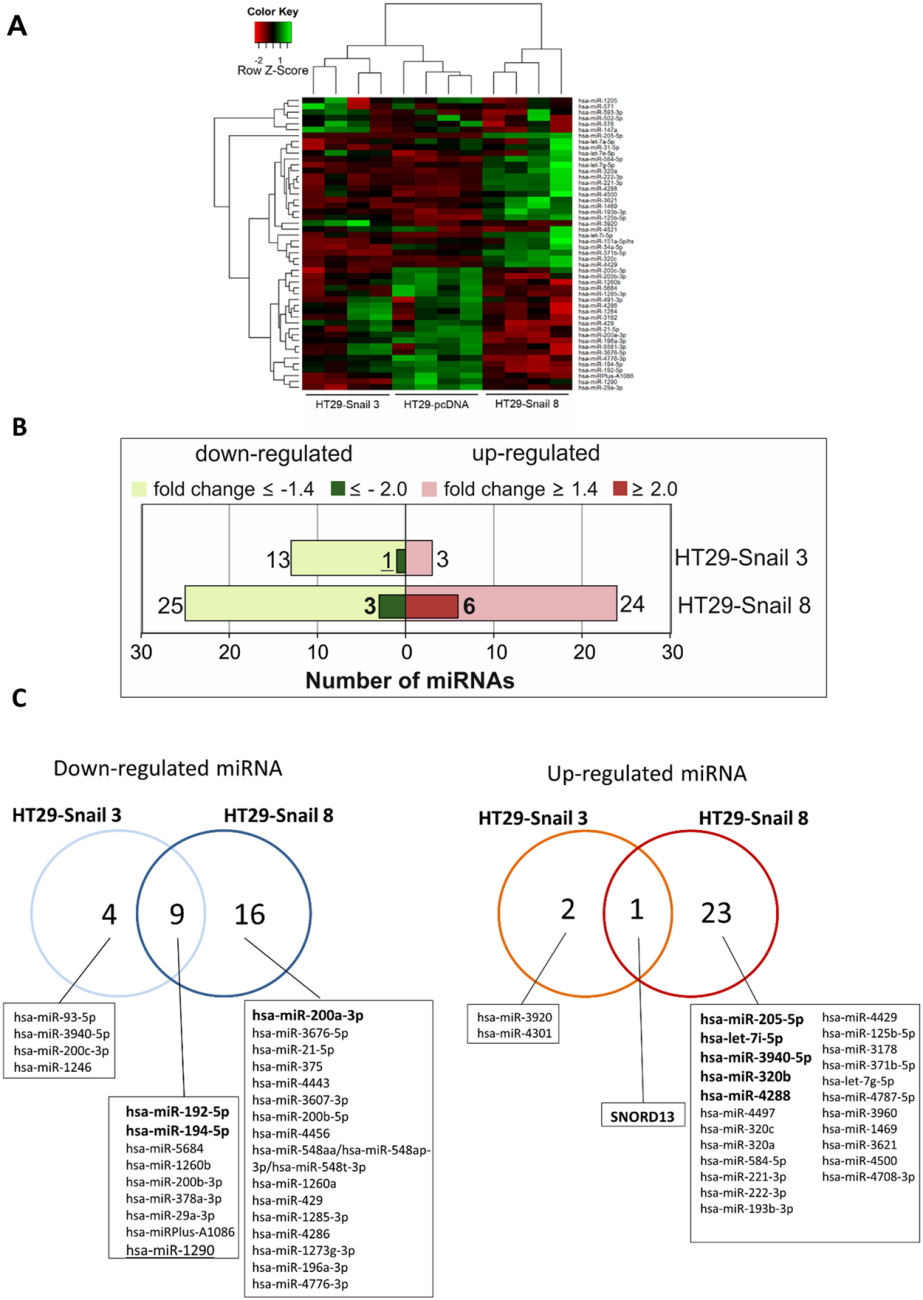
Snail overexpression changes the miRNA expression profile in CRC cells. (**A**) Heat map and unsupervised hierarchical clustering performed on the top 50 miRNAs with the highest standard deviation. The normalized log ratio values were used for the analysis. (**B**) Number of differentially expressed miRNAs detected by microarray analysis, that were either significantly upregulated (red) or downregulated (green) in HT29-Snail-3 and -8 versus HT29-pcDNA. (**C**) Venn diagrams show differentially expressed miRNAs between each clone overexpressing Snail and control cells. The most regulated miRNAs in HT29-Snail-3 and HT29-Snail-8 are marked.

miRNA profiling was performed by microarray analysis of the total mRNA isolated from stable cell lines described above and in^4^. Microarrays included probes targeting all miRNAs and C/D box small nucleolar non-coding RNAs (SNORDs) recently associated with oncogenesis^16^. miRNA profiles were subjected to hierarchical clustering (Fig. 2A). Profiling identified 16 miRNAs differentially expressed in HT29-Snail-3 (13 miRNAs downregulated and 3 miRNAs upregulated) and 49 miRNAs with changed expression in HT29-Snail-8 (25 miRNAs downregulated and 24 miRNAs upregulated). More differences were detected in high- than in moderate-Snail overexpressing clones (Fig. 2B,C; Supplementary Dataset S1 and S2). In the presence of moderately increased Snail, the repression of miRNAs by Snail dominated upregulation (Fig. 2). The presence of high levels of Snail further downregulated miRNAs and upregulation became even more pronounced. A total of 10 small RNAs were commonly altered in both clones; in total, 9 miRNAs were repressed and only one, SNORD13, was upregulated.

At this point we chose miR-192, miR194, miR205, let-7i and SNORD13 for further validation experiments.

### Functional enrichment analysis

**Figure 3:**
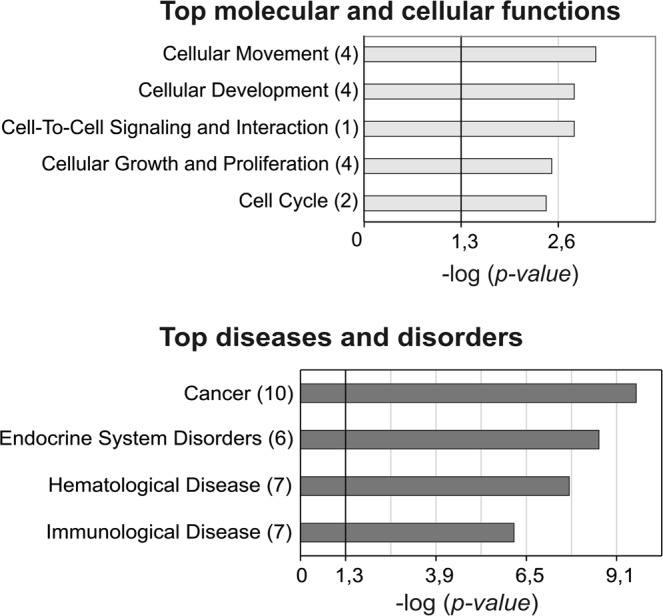
Functional Enrichment Analysis with IPA. The results of microarray analysis interposed onto the database of Ingenuity, with the use of IPA software containing information about miRNA functions. The number of miRNAs involved in the pathways is reported in brackets.

To associate biological functions and diseases with our results and to identify the biological processes that might be triggered in the response to elevate Snail levels, we performed functional enrichment analysis. All differentially expressed miRNAs from HT29-Snail clones and HT29-pcDNA cells were interposed onto the database of Ingenuity using Ingenuity Pathway Analysis software (IPA) containing information about gene functions. After leveraging the differentially expressed miRNA data and complex biological interactions stored in the Ingenuity Knowledge Base, we identified molecular and cellular functions that were significantly altered by Snail overexpression, associated with *cellular movement, cellular development, cell-to-cell signaling, cellular growth and proliferation and cell cycle* (Fig. 3A). Further enrichment analysis showed that deregulated miRNAs (both: upregulated and downregulated) primarily corresponded to miRNA alterations during *cancer* and *endocrine system disorders* (Fig. 3B).

### miRNA expression changes caused by Snail abundance and implication for gene expression in colon cancer cells

**Figure 4:**
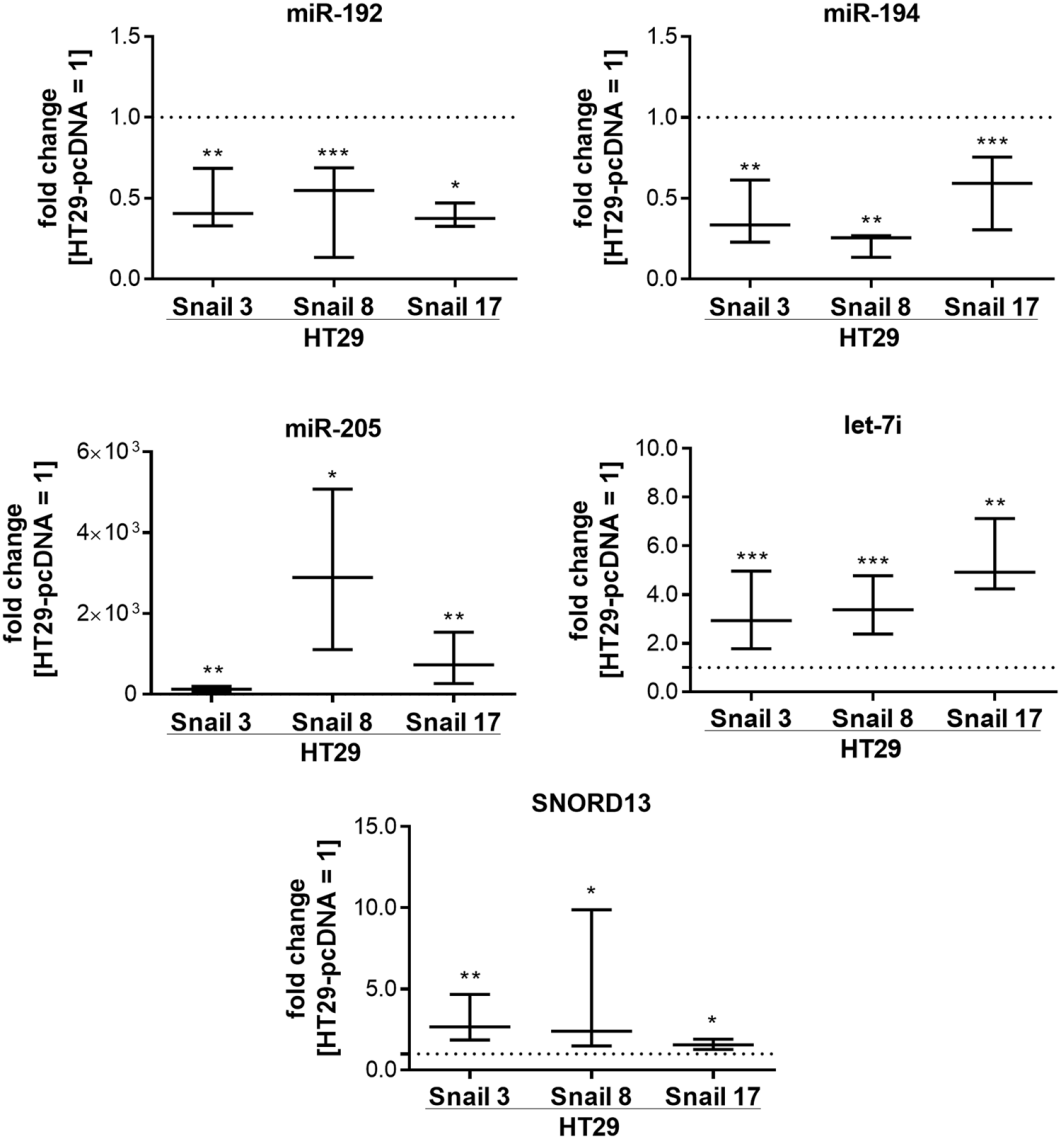
RT- qPCR validation of selected miRNAs in HT29-Snail clones. The results are shown as a median with interquartile range, n ≥ 4, *p ≤ 0,032, **p ≤ 0,016, ***p ≤ 0,004. The results were tested with the Wilcoxon signed-rank test (hypothetical value = 1).

**Figure 5:**
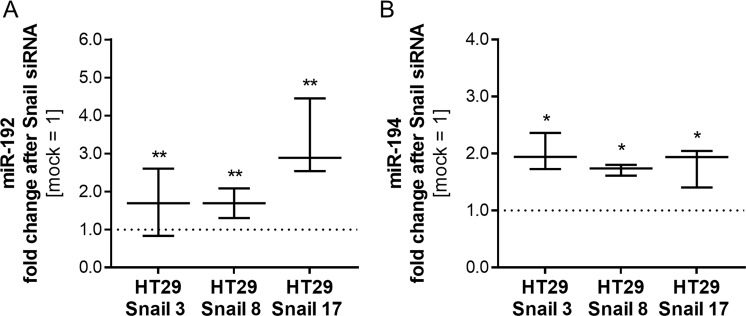
Relative expression of miR-192 and miR-194 after Snail siRNA delivery. The results are shown as a median with interquartile range. The effect of siRNA was tested on 3 different clones and shown as relative to miRNA level in mock cells (not-Snail siRNA-treated HT29-Snail clone). For miR-192, the Wilcoxon signed-rank test was used (hypothetical value = 1), n ≥ 9, **p ≤ 0,008. For miR-194, a one-sample t-test was used (hypothetical value = 1), n ≥ 4, *p ≤ 0,01.

To validate the observed changes in the miRNA profile, the most downregulated miR-192 and miR-194 and the most upregulated miR-205 and let-7i, as well as SNORD13, were examined with real-time quantitative PCR in HT29 clones. Changes detected in miRNA profiling analysis were confirmed (Fig. 4). Furthermore, the effect of Snail silencing on miRNA levels in HT29-Snail clones was tested. The Snail silencing experiments were performed as we described previously^4^. A reduction in Snail mRNA expression and Snail protein levels after Snail siRNA delivery was confirmed (Supplementary Information Fig. S1). Although total silencing of Snail expression controlled by the strong virus CMV promoter was very difficult to achieve, we observed a 50–70% decrease in mRNA expression. A decrease in miR-192 and miR-194 levels in HT29-Snail clones was significantly abrogated after Snail silencing compared to the mock control (control clones not treated with Snail siRNA) (Fig. 5). As expected, the silencing of Snail, a transcriptional repressor, had no effect on the increased production of let-7i, miR-205 and SNORD13 in HT29-Snail clones (Supplementary Information Fig. S2).

Comparison of the up- and downregulated miRNAs from this study with the altered mRNAs in the same cell lines from the previous study^4^ revealed a novel Snail-regulated miRNA/mRNA network. However, it is hard to simply extrapolate the results of such comparison, as a given miRNA may up- or downregulate a large number of mRNAs, and a particular gene may be regulated by diverse miRNAs, whose are tissue- and cell-dependent. Nevertheless, we can point to putative Snail-regulated miRNA/mRNA pathways engaged in early EMT (Table 1 and Supplementary Dataset S3). As an example, our transcriptomic analysis showed that Snail potentiates the expression of thrombospondin (THBS-1), an important protein in the EMT process^4^. All three miRNAs, let-7i, miR-205, and miR-194, have a binding potential to THBS-1 mRNA, but as we also observed upregulation of THBS1 expression in EMT cells, we can conclude that in this model, decreased miR-194 participates in THBS1 expression regulation.

**Table 1:** Top biological targets* of Snail-deregulated miRNAs detected in previously published transcriptomic analysis^4^.

miR-205-5p		average FC
*DNM3*	dynamin 3	−10.951
*SLC4A4*	solute carrier family 4, sodium bicarbonate cotransporter 4	−10.354
*EFCAB4A*	EF-hand calcium binding domain 4A	−4.163
*SHROOM3*	shroom family member 3	−4.103
*CPEB2*	cytoplasmic polyadenylation element binding protein 2	−3.538
*PAQR5*	progestin and adipoQ receptor	−3.52
*ACSL1*	acyl-CoA synthetase long-chain 1	−3.424
*APBB2*	amyloid beta (A4) precursor protein-binding, family B, member 2	−3.39
*NEK6*	NIMA -related kinase 6	−3.329
*RNF213*	ring finger protein 213	−3.296
**let-7i-5p**		
*RCN1*	reticulocalbin 1, EF-hand calcium binding domain	−13.114
*KCTD15*	potassium channel tetramerisation domain containing 15	−12.013
*GPC4*	glypican 4	−9.953
*ONECUT2*	one cut homeobox 2	−8.892
*SPIRE1*	spire homolog 1 (Drosophila)	−8.189
*LGR4*	leucine-rich repeat-containing G protein-coupled receptor 4	−7.034
*PAG1*	phosphoprotein associated with glycosphingolipid microdomains 1	−7.006
*ITGA1*	integrin, alpha 1	−6.452
*BMP2*	bone morphogenetic protein 2	−6.247
*GRAMD1B*	GRAM domain containing 1B	−6.19
**miR-192-5p**		
*SOAT1*	sterol O-acyltransferase 1	9.723
*ALCAM*	activated leukocyte cell adhesion molecule	4.728
*ARL4C*	ADP-ribosylation factor-like 4C	4.211
*ANKRD44*	ankyrin repeat domain 44	3.888
*EGR1*	Early growth response 1	3.281
*PIF1*	PIF1 5′-to-3′ DNA helicase homolog	2.245
*SEPT10*	septin 10	2.134
*XPO4*	exportin 4	2.122
*FGFR1OP*	FGFR1 oncogene partner	2.085
**miR-194-5p**		
*ZC3H12C*	zinc finger CCCH-type 12C	17.251
*PGM2L1*	phosphoglucomutase 2-like 1	12.548
*DUSP10*	dual specificity phosphatase 10	7.924
*FNBP1*	formin binding protein 1	6.916
*FOXF1*	forkhead box F1	6.651
*THBS1*	thrombospondin 1	5.863
*PMEPA1*	prostate transmembrane protein, androgen induced 1	5.823
*MFAP2*	microfibrillar-associated protein 2	4.696
*ZBTB10*	zinc finger and BTB domain 10	4.633
*CFL2*	cofilin 2	4.224

*miRNAs targets determined by TargetScanHuman 7.1 (predicted) and/or miRSearch 3.0 (experimental).

### Snail transcription factor has affinity to the pri-miR-192/194 precursor promoter

**Figure 6:**
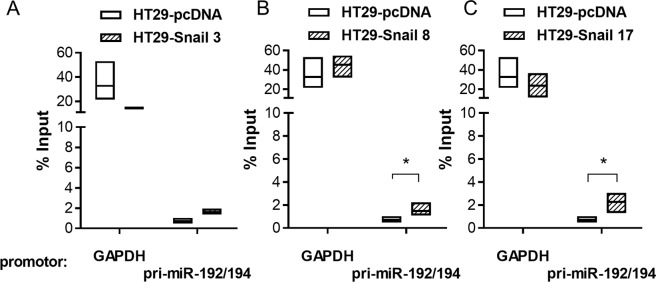
ChIP-qPCR analysis of Snail binding to the pri-miR-192/194 precursor promoter region. Data shown as relative to % input. This includes normalization for both, background levels and input chromatin going into the ChIP. Chromatin immunoprecipitation was performed with the GAPDH promoter binding by anti-RNA polymerase II as a positive control. Differences were tested with the Kruskal-Wallis test followed by Dunn’s multiple comparisons test; n ≥ 4; *p ≤ 0,04

MiR-192 and miR-194 are located on the same cluster on chromosome 11 (11q13.1). Members of the same cluster are usually co-expressed and co-regulated. To determine whether Snail is directly involved in the transcriptional regulation of the pri-miR-192/194 precursor, we performed putative promoter analysis. After the identification of possible promoter regions with Gene2Promoter (Genomatix) we used MatInspector (Genomatix) to selected region analysis. This enabled us to find possible transcription factor binding sites. Among the twenty prospective transcription factors identified, using a stringent matrix similarity score cutoff of 0.95, Snail was predicted to bind to E-box gcgcCAGGtgt with p = 0.03 (Supplementary Dataset S4). The ability of Snail to bind to the pri-miR-192/194 promoter region was further examined using a ChIP assay with an anti-Snail antibody. We observed a significant increase in Snail binding to the identified promoter region in HT29 clones with high Snail overexpression levels when compared to the control clone HT29-pcDNA (Fig. 6). In clone 3 with moderate Snail overexpression, the trend was comparable, but the differences did not reach statistical significance.

### Snail affected miRNA content in extracellular vesicles (EVs)

Since EVs are the key components of intercellular communication in CRC (and other cancers as well)^17,18^, we investigated how Snail affects the miRNA content of EVs released from HT29 cells. We used purified and characterized EVs (as described in the Methods section and conference communications^19,20^) for mRNA isolation and miRNA detection by next-generation sequencing (NGS). Analysis of the cargo of EVs released from clones overexpressing Snail showed an increase in miR-205 and let-7i and a decrease in miR-192 and miR-194 levels when compared to EVs released from HT29-pcDNA cells (Table 2). For these specific miRNAs, the pattern of intracellular expression changes is reflected in the EVs content (Table 2).

**Table 2:** Snail deregulated miRNA levels in EVs released from HT29 cells; n = 3, p-value ≤ 0.003.

miRNA name	Cells HT29-Snail vs HT29-pcDNA	Extracellular vesicles HT29-Snail vs HT29-pcDNA (fold change)		
		HT29-Snail-3	HT29-Snail-8	HT29-Snail-17
miR-205	Upregulated	81.01	2368.90	342.51
let-7i		2.67	3.03	3.18
miR-192	Downregulated	−1.59	−2.14	−2.51
miR-194		−1.80	−2.44	−2.75

## Discussion

For a long time, EMT was perceived as a single transition between epithelial and mesenchymal states. However, since the discovery of the process, there have been many observations, including our previous studies^4^, which documented the so-called “partial” EMT phenomenon. Now EMT must be perceived as a dynamic transition within the spectrum of intermediate states with metastable points in the process^21^. EMT was always regarded as a promising therapeutic target in cancer^22^. There have been many attempts to remove more invasive cells that undergo EMT or to revert the transition of metastable cells. The most auspicious approach, in light of current knowledge and our observations, seems to be the interference in cancer cell plasticity rather than targeting cells with ever-shifting phenotypes^23^. A major challenge in this attempt is to understand the molecular basis of cancer cell plasticity and pathways activated at the beginning and throughout the EMT process. To choose successful therapy, scientific groups tend to identify drivers and fine-tuners of epithelial–mesenchymal plasticity or use available data to create statistical models to describe the EMT spectrum with algorithms^24^. Nevertheless, cancer cell aggressiveness is strictly tissue- and subtype-specific and must be considered during therapy selection.

Knowledge concerning changes in miRNA regulation at the early step of EMT in CRC remains elusive. To shed more light on this important process we focused here on miRNA regulation by the transcription factor Snail in the context of colon cancer cells. We have broadened our previously published transcriptomic data^4^ with miRNA profiling. Investigation of miRNA and/or mRNA expression in CRCs at various stages of the disease and differences between tumour and normal tissues have been conducted by various groups^25–28^. However, to date, no study has shown the effect of Snail on the rearrangement of the miRNA profile of cancer cells during the early steps of EMT.

There are data indicating that Snail stability and availability in the nucleus are crucial for its action, which is regulated at different levels and not exclusively by transcription control^29^. We hypothesized that Snail overexpression under a strong virus promoter (CMV) achieved by the stable transfection of HT29 may, to some extent, overcome these regulatory processes. Snail clones grew more scattered, acquired a spindle-shaped morphology, lost cell-to-cell contact and migrated more eagerly. We observed downregulation of tight junctions, adherence junctions and basement membrane adhesion components, but these changes were not proportional to the Snail overexpression level in all cases^4^.

Here we show that overexpression of Snail in CRC cells does not change the global efficiency of miRNA processing, as was observed under particular conditions such as hypoxia^14^. Nevertheless, Snail significantly triggers changes in individual miRNA levels. The performed array analysis showed deregulation from 16 to 49 miRNAs in HT29 clones expressing various levels of Snail. MiR-205, let-7i and SNORD13, which was included in the array, were found to be particularly overexpressed, while miR-192 and miR-194 were the most reduced. We have successfully validated these changes. IPA analysis showed that the observed miRNA alterations resemble those associated with *cellular movement* and *cancer*. This observation is in agreement with the migratory phenotype previously observed by our group for Snail overexpressing clones^4^ and suggests that Snail can influence the mechanism of cell movement by affecting miRNAs.

The significant upregulation of miR-205 and let-7i in HT29-Snail cells seems to be controversial as they are widely viewed as tumour suppressors. However, if we look into details, the differences in reports about miRNA functions may be conditional on the specific tumour and its microenvironment^30,31^.

MiR-205 is deregulated in different cancer types and subtypes and differs in functions. MiR-205 either inhibits proliferation and invasion or facilitates tumour initiation and proliferation as an oncogene (reviewed by Cao S. *et al*.^30^). When analysed in CRC tissues, it was found to be differently deregulated^32^. In our model, miR-205 is consistently upregulated in EMT regulated by Snail in HT29 cells, which corresponds to recent suggestions that it contributes to aggressive behavior, as reported for mucinous CRC^33^. We also detected more miR-205 in extracellular vesicles released by HT29-Snail cells (Table 2). This result is interesting since it was shown that conditioned medium from cancer cells overexpressing miR-205 stimulated angiogenesis of the endothelial cells^34^. Interplay between cancer cells, endothelial cells and macrophages in the tumour microenvironment was reported to be essential for intravasation^35,36^. Potentiated miR-205 expression was correlated with one of its target DNM3 mRNA decrease (Table 1). Dynamin 3 (DNM3), considered as a cancer suppressor^37^ and, from the tumour point of view, redundant during disease progression, was decreased in HT29-Snail cells (Table 1).

Let-7 family upregulation during cancer progression is not frequently observed as they are also considered to be tumour suppressors^31^. However, a higher level of plasma let-7 family and poorer disease prognosis have been correlated in patients with colorectal and other types of cancers^38–41^. In our model, we observed an increased level of let-7i in HT29-Snail clones and deregulation of its target genes (Table 1 and Supplementary Dataset S3). Its level was also increased in HT29-Snail extracellular vesicles. Protection of endothelial cells through let-7i delivered by EVs to endothelial cells in the tumour microenvironment would be beneficial for cancer progression, but at this point it is only a hypothesis based on the reported protective role of let-7i on brain endothelial cells in oxygen-glucose deprivation conditions^42^. That hypothesis needs to be carefully evaluated. Nevertheless, changes in the extracellular vesicles cargo regulated by Snail may modify communication between CRC and neighbouring cells at the very beginning of EMT.

In addition to miRNAs, our data provided an argument for the evaluation of the importance of snoRNAs (small nucleolar RNAs) in colorectal cancer progression and metastasis. Interestingly, snoRNAs were shown to be processed into small RNAs with miRNA functions^43^. We observed and validated the increase in SNORD13, a member of the snoRNAs family associated with the production of cell protein synthesis machinery in our early EMT model. SNORDs are not the main subject of this study but as a side observation, their upregulation is in agreement with the growing role of non-coding RNAs in EMT^44^. Our results correlate with the striking difference in snoRNA expression between normal and malignant cells^16,45^. These changes may have consequences for mRNA translation and EMT progression in CRC, as was already observed for NSCLC in which invasion was promoted by SNORD78^46^.

While searching for miRNAs potentially repressed through Snail activation, we compared our results with miRNA expression signature analyses of three previously published datasets of epithelial and mesenchymal NCI60 cancer cell lines^47–49^. Eleven different miRNAs that were decreased in our model (among them miR-192 and miR-194) were also downregulated in colon cancer cell lines that were defined as more mesenchymal (Supplementary Information Table S1). The most downregulated miRNAs, miR-192 and miR-194, are located on the same cluster on chromosome 11 (11q13.1). miR-194 is also located on the cluster with miR-215 on chromosome 1 (1q41) but we did not observe fluctuations in miR-215 expression. miR-192 and miR-194 are described as suppressors of metastasis in many types of cancer^50,51^. Their expression was inversely correlated with the metastatic potential of colon cancer cells^52,53^. There are speculations that miR-192 and miR-194 suppressive functions in CRC are related to cell cycle control or inhibition of invasiveness, and it is suggested that restoration of its expression could have therapeutic potential^51,54^. We observed that the levels of downregulated miR-192 and miR-194 by Snail in HT29 cells were partially rescued by the silencing of Snail. We also detected Snail affinity for the pri-miR-192/194 promoter region with chromatin immunoprecipitation. This result showed for the first time a role of Snail, an EMT inducer, in the regulation of pri-miR-192/194 precursor transcription in CRC. In light of the reported reciprocal SLC39A6/Snail pathway regulation by miR-192^51^, further investigations of another feedback loop regulating EMT in CRC are needed.

To the best of our knowledge, this study provides evidence for the first time that Snail, as an initial transcription factor driving colon cancer cell EMT, modulates specific miRNA expression. In the case of the most deregulated miRNAs, it is possible that Snail interacts with miRNA precursor promoter regions or stimulates miRNAs transcription through yet unknown mechanisms. Moreover, reported changes in miRNA profiles induced by Snail are reflected in gene transcription changes and in the extracellular vesicles content released from CRC cells.

Thus, we show that Snail modulates the EMT process at early steps not only through gene but also by miRNA regulation and may also affect cancer cell communication with other cells within the tumour microenvironment through EV cargo modification. In light of our observations, statements about miR-205 and let-7i as tumour suppressors in CRC have to be revised as their transcription levels seem to be dependent on the cancer cell phenotype.

## Materials and Methods

### Cell culture and reagents

The HT29 cell line (cells: colon, disease: colorectal adenocarcinoma) from ATCC (Manassas, VA, USA) was cultured in McCoy’s 5A Medium (Thermo Fisher Scientific, Waltham, MA, USA), supplemented with 10% FBS (Thermo Fisher Scientific) with antibiotics, streptomycin, penicillin (Sigma-Aldrich, St. Louis, MO, USA), and primocin (InvivoGen, San Diego, CA, USA), in a 90–95% humidified atmosphere with 5% CO_2 _. HT29-Snail clones generated as described previously^4^ were cultured with 200 μg/mL G418/Geneticin (Gibco/Thermo Fisher Scientific). Cells were tested monthly for mycoplasma (PlasmoTest, InvivoGen). The HT29 cell line and HT29-pcDNA control clone were authenticated by ATCC using the Short Tandem Repeat (STR) analysis.

### RNA isolation and real-time quantitative PCR analysis of genes and miRNA expression

Total RNA was isolated with the miRCURY^TM^ RNA Isolation Kit (Exiqon, Vedbæk, Denmark) according to the manufacturer’s instructions. The quality control of RNA was performed using the 2100 Bioanalyzer (Agilent Technologies, Palo Alto, CA, USA). One microgram of total RNA from cells (RIN ≥ 9) was reverse transcribed using the High Capacity cDNA Reverse Transcription Kit or TaqMan™ MicroRNA Reverse Transcription Kit (Applied Biosystems, Foster City, CA, USA). TaqMan Gene Expression Assays (Thermo Fisher Scientific): Drosha (Hs00203008_m1) and Dicer (Hs00229023_m1) or TaqMan microRNA Assays (Thermo Fisher Scientific): hsa-miR-205 (#000509), hsa-miR-194 (#000493), hsa-miR-192 (#000491), hsa-miR-181a (#000480), hsa-let-7i (#002221) or RT² qPCR Primer Assay for Human SNORD13 (Qiagen, Hilden, Germany) were further used for transcript quantification through real-time quantitative PCR using the TaqMan Universal PCR Master Mix or Power SYBR Green PCR Master Mix and the ABI Prism7900-HT Detection System (Applied Biosystems). A standard PCR cycle was the following: incubation at 50 °C for 2 min, 95 °C for 10 min, followed by 45 cycles of 95 °C for 15 sec and 60 °C for 1 min. GADPH and β-actin mRNA transcripts were used as internal control genes and hsa-miR-181a was used as internal control miRNA. The amount of target in the various samples was calculated using the 2^−ΔΔCT^ or the 2^−ΔCT^ relative quantification method with DataAssist v.3.01.

### Screening of miRNA expression

miRNA screening was conducted at Exiqon Services. A total of 750 ng total RNA from both the sample and reference was labelled with Hy3™ and Hy5™ fluorescent labels, respectively, using the miRCURY LNA™ microRNA Hi-Power Labeling Kit, Hy3™/Hy5™ (Exiqon) following the procedure described by the manufacturer. The Hy3™-labelled samples and aHy5™-labelled reference RNA sample were mixed pair-wise and hybridized to the miRCURY LNA™ microRNA Array 7th Gen (Exiqon), which contains capture probes targeting all miRNAs for human, mouse or rat registered in the miRBase 18.0 as well as miRNA from viruses and SNORDs. The hybridization was performed according to the miRCURY LNA™ microRNA Array instruction manual using a Tecan HS4800™ hybridization station (Tecan, Austria). Microarray slides were scanned and stored in an ozone-free environment until scanned using the Agilent G2565BA Microarray Scanner System (Agilent Technologies). The image analysis was carried out using ImaGene® 9 (Exiqon). The quantified signals were background corrected (Normexp with offset value 10^55^) and normalized using the global LOWESS regression algorithm. The background threshold was calculated for each individual microarray slide as 1.2 times the 25th percentile of the overall signal intensity of the slide. miRNAs with intensities above the threshold in less than 20% of the samples were removed from the final dataset. A total of 1679 probes were discarded by this filtering procedure. The results were interposed onto the database of Ingenuity using Ingenuity Pathway Analysis software (Ingenuity®Systems, Redwood City, CA, USA; http://www.ingenuity.com) containing information about the miRNAs functions. IPA analysis was performed with the collaboration of the Core Laboratory for Microarray Analysis (IBB, PAS, Warsaw, Poland).

### HT29 nucleofection, Snail silencing with siRNA

HT29 cells were grown to 85% confluence and siGENOME SNAI1 siRNA and non-targeting NT-siRNA, 60 pmol/10^6^ cells (Dharmacon, Lafayette, CO, USA) were delivered to HT29-Snail clones using Amaxa® 4D nucleofector® X Unit (Lonza, Basel, Switzerland). A mixture of 4 siRNAs provided as a single reagent in a SMARTpool format was used. 72 h post-nucleofection cell lysates were collected and analysed.

### Extracellular vesicle purification and miRNA content analysis

Cells were grown to 70–80% confluence, washed with RPMI 1640 medium and cultured in serum-free RPMI 1640 supplemented with  streptomycin, penicillin (Sigma-Aldrich). After 24 h conditioned medium (CM) was collected and centrifuged at 350 × g for 10 min, then at 2 000 × g for 20 min. Fractions containing extracellular vesicles (EVs) were obtained after ultracentrifugation (1.5 h at 100 000 × g). The pellets were treated with RNase A (20 μg/ml) (30 min, RT) to avoid possible non-vesicular nucleic acids bound to the external surface of EVs. Next, EV fractions were washed in PBS, and a second ultracentrifugation was performed (1.5 h at 100 000 × g). All centrifugations were performed at 4 °C. Purified EVs were quantified for vesicle number by NanoSight analysis (mean of concentration values [particles/ml]: 1.3e+11), and the purity of EVs was confirmed by Western blot analysis (presence of tetraspanin CD63, absence of cytochrome c).

Total RNA was isolated with the miRCURY^TM^ RNA Isolation Kit (Exiqon) according to the manufacturer’s instructions. The quality control of RNA was performed using the 2100 Bioanalyzer (Agilent Technologies, Palo Alto, CA, USA). Next-generation sequencing (NGS) analysis of the miRNAs identified in the RNA samples isolated from EVs was performed by Exiqon (https://www.exiqon.com/small-rna-ngs). Briefly, NGS sequencing libraries were prepared, quantified and sequenced from the EV RNA samples. The collected reads were subjected to quality control, alignment and differential expression analysis, which identified a subset of the microRNAs that had significant differences in the counts between the experimental groups.

### Promoter analysis

First, we identified a possible promoter region with Gene2Promoter. The DNA sequence of the 2.0 kb pri-miR-192/194 potential promoter was analysed with MatInspector (http://www.genomatix.de)^56^ that identifies putative transcription factor binding sites with weight matrices representing consensus recognition sequences for different transcription factors defined in the MatInspector Library: Matrix Family Library Version 10.0 (October 2016).

### Chromatin Immunoprecipitation

Chromatin immunoprecipitation (ChIP) was performed using the EZ-ChIP kit from Millipore (Billerica, Massachusetts, USA) according to the manufacturer’s protocol. Human anti-Snail antibody (R&D, Minneapolis, MN, USA), normal mouse IgG and anti-RNA polymerase II were used for immunoprecipitation. For the detection of the pri-miRNA promoter region, we used primers (5′-TTTATGAGGCCGATTTGGGGT-3′ and 5′-CCCAGGTCCATGGTCTTTTC-3′) specific for a 123 bp region in the pri-miR-192/194 putative promoter region that encompasses the potential Snail binding site (MatInspector/Genomatix software). As a positive control, we used primers for the detection of the CDH-1 (*E-cadherin)* promoter^57^. Real-time quantitative PCR amplification of soluble chromatin prior to immunoprecipitation was used as an input control. The ChIP-real time quantitative PCR data were analysed according to the Percent Input Method (Thermo Fisher Scientific). Signals obtained from the ChIP were divided by signals obtained from an input sample.

### Statistical analysis

The Shapiro-Wilk test was used to confirm the Gaussian distributions of raw data. Data non-departing from normal distribution are presented as the mean and SD; otherwise, medians and interquartile ranges are used. For the unpaired comparisons, the appropriate Student’s t test (or the Welch’s test for unequal SDs) was performed to test the differences between groups for normally distributed data. The Mann-Whitney U test was performed to test the differences between groups of data with non-normal distributions. In the case of multiple comparisons, data departing from normal distribution differences were tested with the Kruskal-Wallis test followed by Dunn’s multiple comparisons test. In the case of relative comparisons to hypothetical value, the Wilcoxon signed-rank test or one-sample t-test were used according to the data distribution.

## Supplementary information

Supplementary Dataset S1

Supplementary Dataset S2

Supplementary Dataset S3

Supplementary Dataset S4

Supplementary information
