# Supplementary material for: Regulation of miRNAs by Snail during epithelial-to-mesenchymal transition in HT29 colon cancer cells
Source: Sci Rep. 2019 Feb 15;9:2165. doi: 10.1038/s41598-019-39200-7 (PMC6377707; doi:10.1038/s41598-019-39200-7)
Supplement: Supplementary file 5 — Supplementary information [file 41598_2019_39200_MOESM5_ESM.pdf]

Regulation of miRNAs by Snail during epithelial-to-mesenchymal transition in HT29 colon cancer cells.

Przygodzka Patrycja*, Papiewska-Pajak Izabela, Bogusz Helena, Sochacka Ewelina, Boncela Joanna, Kowalska M. Anna

Supplementary Information

Figure S1A

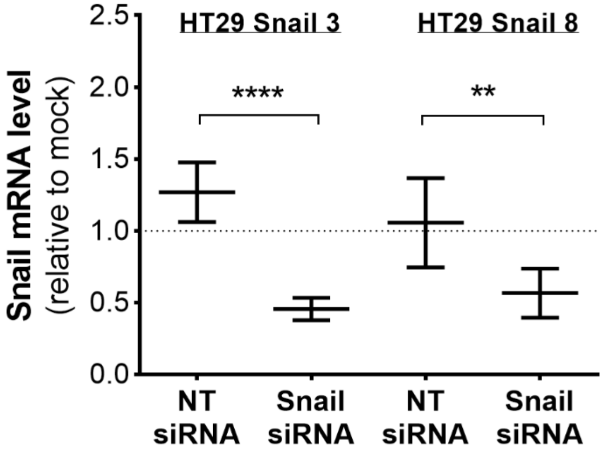

Supplementary Figure S1A. *Snail silencing effects through Snail-siRNA delivery.* Relative Snail mRNA expression after Snail siRNA delivery. Results shown as mean with SD. Differences where tested with unpaired t test with Welch's correction **p≤0,003; ****p<0,0001; n≥6.

Figure S1B

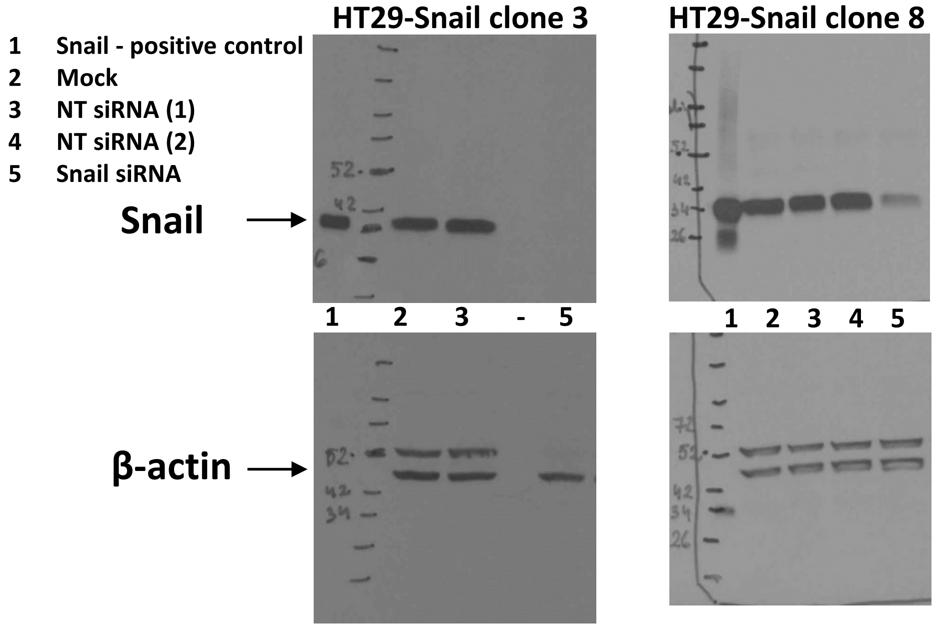

Supplementary Figure S1B. *Snail silencing effects through Snail-siRNA delivery.* Snail protein was analyzed by immunobloting. Images show representative results of Western blot analysis. Bands were quantified by densitometry. Intensity of Snail band was normalized against respective β-actin band. Results shown as mean with SD. Differences were tested with t test with Welch's correction; **p=0,0072; *p=0,03; n=3.

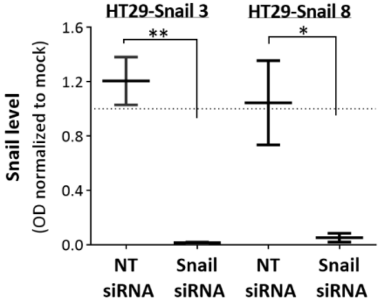

Figure S2

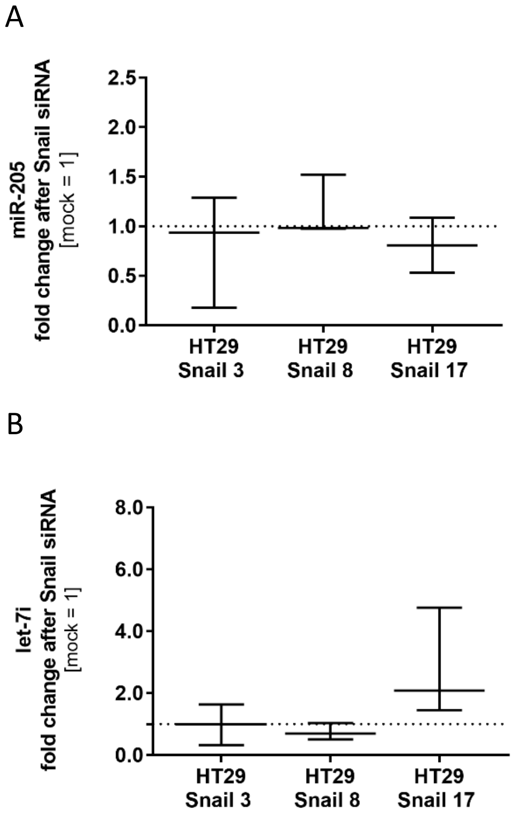

Supplementary Figure S2. *Relative miRs expression after Snail silencing.* Results shown as median with interquartile range. The effect of siRNA shown as relative to miRs level in mock. Results were tested with Wilcoxon signed-rank test, $n \geq 3$.

Supplementary Table S1

List of miRNAs found downregulated in mesenchymal vs epithelial, (NCI60 panel) in the large-scale analysis of miRNA expression signatures and in the intermediate epithelial vs epithelial, in the HT29-Snail clones.

**miRNA**	**average LogFC** HT29-Snail (clones 3 and 8) vs HT29-pcDNA
hsa-mir-129	-0,329
hsa-miR-136	-0,085
hsa-miR-141	-0,337
hsa-miR-155	-0,256
hsa-miR-200b	-0,614
hsa-miR-200c	-0,506
hsa-mir-331	-0,328
hsa-miR-378	-0,554
hsa-miR-429	-0,278
hsa-mir-192	-1,051
hsa-mir-194	-1,162
